# Health-Risk Behavior-, Mental Health-, and Physical Exercise-Related Risk Factors for Tooth Fractures in Korean Adolescents

**DOI:** 10.3390/ijerph17217815

**Published:** 2020-10-26

**Authors:** Han-Na Kim, Yong-Bong Kwon, Eun-Joo Jun, Jin-Bom Kim

**Affiliations:** 1 Department of Dental Hygiene, College of Health and Medical Sciences, Cheongju University, Cheongju 28503, Korea; hnkim@cju.ac.kr; 2 Department of Preventive and Community Dentistry, School of Dentistry, Pusan National University, Yangsan 50612, Korea; naeda1103@naver.com (Y.-B.K.); jsilver@pusan.ac.kr (E.-J.J.); 3 BK21 FOUR Project, School of Dentistry, Pusan National University, Yangsan 50612, Korea

**Keywords:** adolescent, depression, health behavior, physical exercise, tooth fracture

## Abstract

We aimed to determine factors related to tooth fracture experience in Korean adolescents. This study used data from the 14th Korea Youth Risk Behavior Survey in 2018, a cross-sectional web-based survey of health-risk behaviors among a representative sample of Korean middle- and high-school students aged 12–17 years. A total of 60,040 participants were selected using a complex sampling design of the survey from 400 middle schools and 400 high schools. They answered a self-administered questionnaire survey in classrooms. Explanatory variables included those pertaining to health-risk behaviors, mental health, and physical exercise. Complex-sample multivariable logistic regression models were applied to identify factors related to tooth fracture experience in the past 12 months. The overall prevalence of dental fracture experience was 11.4%. Risk factors related to tooth fractures in Korean adolescents were unhealthy behaviors such as alcohol and tobacco consumption; mental health problems including stress, depression, and suicidal ideation; and intensive physical exercise. The major risk factor related to tooth fractures was depression. To prevent tooth fractures among adolescents, schools should strengthen mental health education, encourage mouthguard use during intensive physical exercise, and develop school environments to prevent orofacial injuries. Further studies on various risk factors related to tooth fractures are warranted.

## 1. Introduction

With advancements in transportation, expansion of living spaces, and greater travel and sporting activity worldwide, trauma risk due to accidents has been increasing [1,2]. Head and neck trauma is often accompanied by dental trauma, and in a comprehensive literature search of studies published in 1996–2016 the prevalence of traumatic dental injuries in the permanent dentition worldwide was found to be 15.2% for 12-year-old subjects [3].

Adolescence is a period of rapid growth and a very active period with a high incidence of dental trauma. Dental trauma has been reported in 16.4% of children aged 16 years in western Norway [4], 35.0% of those aged 11–13 years in Thailand [5], and 43.8% of 14-year-olds in Newham, London, UK [6]. In the National Oral Health Survey conducted by the Ministry of Health and Welfare in Korea in 2010, the dental trauma experience rate was reported to be 17.7% among 12-year-olds and 19.3% in 15-year-olds [7,8]. Among 14–16-year-olds in Yangsan, Korea, the rate of dental trauma was reported to be 16.8% [9].

Visible signs of dental trauma can range from a simple chip of the enamel to a severely fractured and displaced tooth, or even complete loss of the tooth from either avulsion or subsequent complications. Although tooth displacement or dislocation have been reported to occur with dental trauma of deciduous teeth, tooth fracture is known to occur more easily in permanent teeth than in deciduous teeth [10,11]. In a survey of patients who visited dental clinics in Korea, approximately half of the cases with dental trauma in the permanent dentition were reported to show tooth fractures [12]. Similarly, a survey of 14–16-year-olds in Yangsan reported a high rate of tooth fracture (88.3%) among those with dental trauma [9].

Dental trauma including tooth fracture and tooth loss have also been investigated in the 2010 National Oral Health Survey conducted by the Ministry of Health and Welfare [7]. In the questionnaires of the Korea Youth Risk Behavior Survey (KYRBS) of the Ministry of Education and the Korea Centers for Disease Control and Prevention (KCDC), only tooth fractures were investigated [13]. KYRBS has been researching the status and trend of health behaviors among middle- and high-school students every year since 2005 in order to establish a plan for health promotion for adolescence. In addition to health behaviors, this survey included assessments of physical exercise, mental health, oral disease symptoms, and tooth fracture [7]. Perheentupa et al. [13] reported that mental distress and a history of previous injuries were associated to increase the risk for dental injuries.

The purpose of this study was to analyze risk factors, such as health behaviors, mental health issues, and physical exercise patterns related to tooth fracture among adolescents by using the 14th 2018 KYRBS data for middle- and high-school students. The hypotheses of this study are as follows. (1) Adolescents with health-risk behaviors will have a higher dental fracture experience than those without health-risk behaviors. (2) Adolescents suffering from mental health will have high dental fracture experience. (3) Adolescents performing intensive physical exercises will have high tooth fracture experience.

## 2. Materials and Methods

### 2.1. Study Participants

This study used data from the 14th KYRBS conducted in 2018. This survey is an anonymous self-administered online survey conducted to analyze health-risk behaviors of middle-school and high-school students in South Korea. These data were requested from the KCDC’s official website of KYRBS and downloaded after obtaining approval from the relevant institution [14]. The survey, a government-approved survey conducted on the basis of Article 14 of the Law for the Promotion of the Nation’s Health, has been conducted annually since 2005 by the Ministry of Education, the Ministry of Health and Welfare, and the KCDC [14,15].

The 14th KYRBS conducted in 2018 was a government-approved statistical survey (approval no. 117058) designed to represent Korean middle- and high-school students nationwide. In the sample distribution stage, the sample size was set to 400 middle schools and 400 high schools. The regional divisions were categorized in terms of city, province, city scale (large, small, and medium city, and county area), and regional county. The number of sample schools was calculated on the basis of the ratio of males and females in middle schools and the ratio of males and females and educational characteristics in high schools. Thus, a total of 62,823 students from 400 middle schools and 400 high schools were designated as the study sample, and 30,463 male students and 29,577 female students actually participated in the survey. The participation rate was 95.6% [14]. Participants from schools designated within the sample were randomly assigned to one computer per school computer room with internet access to answer the questionnaires.

Questionnaires were developed through national and international data and expert advisory committees related to youth health. The survey included 103 questions across 15 domains, which were smoking, alcohol consumption, physical exercise, diet, obesity and weight control, mental health, prevention of accidental injury, oral health, personal hygiene, sexual behavior, atopy and asthma, drugs, internet addiction, health equity, and violence.

### 2.2. Variables

In the questionnaires, occurrence of a tooth fracture was a dependent variable. For this study, the relationship of survey variables in the domains of alcohol drinking, smoking, mental health, and physical exercises with tooth fracture experience were selected as the analysis target (Table 1).

Health-risk behaviors among independent variables included alcohol consumption and smoking. For the alcohol intake domain, mean alcohol intake volume and the frequency of binge drinking experiences in the last 30 days were selected, while the amount of smoking in the last 30 days was selected in the smoking domain. In the mental health domain, we selected the level of usual stress perception in the last 7 days, the level of satisfaction in fatigue recovery by sleep in the last 7 days, and the experience of depression and suicidal thoughts in the last 12 months. For physical exercise, days of physical exercise for more than 60 min, days of intense physical exercise, days of muscle strength training, and the number of school gym classes in the last 7 days were selected. Categorizations of each variable are presented in Table 2.

### 2.3. Statistical Analysis

The collected data were analyzed by using an IBM SPSS 25.0^®^ (IBM Corp., New York, NY, USA) to analyze the relationship between tooth fracture experience and explanatory variables by complex-sample chi-square test and complex-sample multivariable logistic regression analysis.

In the sampling process, KYRBS stratified the population to minimize sampling errors, divided regions and schools into strata using stratification variables, and when there was only one colony in the strata, integrated it with the adjacent sample design layer. Since the integrated sample design strata is disclosed in the raw data, the sample design strata variable should be used as the stratification variable in data analysis [13]. Accordingly, in the KYRBS analysis, it is essential to use the analysis of complex samples represented by weights based on the population. Therefore, after the planning file was created using stratified variables (kstrata), colony variables (cluster), and weighted variables (w), complex-sample analysis was performed. The results of the analysis were expressed as estimated percentages, odds ratios (ORs), and 95% confidence intervals (CIs), and the significance level was determined as type I error 0.05.

## 3. Results

The tooth fracture experience rate was 11.4% in all adolescents, 11.8% in males, and 11.1% in females, with the experience rate being higher in males (*p* = 0.023) and increasing with the fracture grade (*p* = 0.009) (Table 3). 

The tooth fracture experience rate was 10.7% among adolescents who had never consumed alcohol before and 20.4% among those with an average alcohol intake of 8 bottles or more in the last 30 days, indicating that higher average drinking volumes corresponded to a higher tooth fracture experience rate (Table 2). The tooth fracture experience rate was 10.7% in adolescents who had never smoked and 22.9% in adolescents who had smoked more than 20 cigarettes per day in the last 30 days. Thus, the experience rate increased with the number of cigarettes smoked daily (Table 2).

The tooth fracture experience rate among adolescents who perceived “so much” stress on a daily basis was 14.3%, while it was 10.9% in adolescents who perceived no stress at all. Thus, a higher than usual stress perception corresponded to a higher rate of tooth fracture experience.

The tooth fracture experience rate was 10.7% in adolescents with very good fatigue recovery through sleep in the last 7 days and 14.0% in those with insufficient fatigue recovery by sleep. Thus, the rate of tooth fracture experience increased as the level of fatigue recovery by sleep decreased (Table 2).

The tooth fracture experience rate was 10.3% in adolescents who had never felt depressed and 14.5% in those who felt depressed enough to stop daily life for two weeks in the last 12 months. The rate of tooth fracture experience was higher in adolescents who experienced depression. Similarly, the tooth fracture experience rate was 11.0% in adolescents who had never experienced suicidal ideation in the past 12 months, and 14.7% in those who had experienced suicidal ideation. The rate of tooth fracture experience was higher in adolescents who experienced suicidal ideation (Table 2).

The tooth fracture experience rate was 10.3% in adolescents who had never performed physical exercise for more than 60 min a day in the last 7 days and 15.1% in those who had performed prolonged exercise for 7 days. Thus, the rate of tooth fracture experience increased with the number of days involving prolonged physical experience. The tooth fracture experience rate was 10.6% in adolescents who did not perform intense physical exercise during the last 7 days and 13.6% in adolescents who had performed intense physical exercise for 5 or more days over the past week. Thus, the rate of tooth fracture experience increased with the number of days on which the participants performed intense physical exercise. The tooth fracture experience rate was 10.5% in adolescents who performed no muscle strength exercise over the last 7 days, 13.6% in adolescents who performed muscle strength exercise for 5 or more days, and 12.1% in adolescents who performed school gym classes for 3 or more days. Thus, the tooth fracture experience rate increased with the number of days of muscle strength exercise (Table 4).

Table 5 shows the adjusted association of tooth fracture experience related to health-risk behaviors by logistic regression model.

In Model 1 adjusted for sex and grade, the variables of alcohol intake volume, binge drinking, smoking, stress perception, fatigue recovery level by sleep, depression, and suicidal ideation were related to tooth fracture experience. In Model 2, all related variables were adjusted, and the variables of sex, alcohol intake volume, smoking, stress perception, fatigue recovery level by sleep, depression, and suicidal ideation were associated with tooth fracture experience.

Model 2 confirms the relationship between tooth fracture and health-risk behaviors and mental and physical status. Male students showed a higher tooth fracture experience rate than female students. Tooth fracture experience was higher in participants who consumed more alcohol, had frequent binge drinking experiences, and smoked more cigarettes. Tooth fracture experience rate also increased with higher stress perception, lower fatigue recovery level by sleep, depression, and suicidal ideation (Table 5).

In Model 1, which was adjusted by only sex and grade, the number of days of exercise over 60 min a day, the number of days of intense physical exercise, and the number of days of muscle strength exercise were related to tooth fracture experience. In Model 2, grade, intense physical exercise, and muscle strength exercise were associated with tooth fracture experience. Model 2 examined the relationship between physical exercise and tooth fracture, and adolescents with advanced grades, intense physical exercises days, and many muscle strength exercises had a higher tooth fracture experience (Table 6).

## 4. Discussion

Participants in the 14th KYRBS were aged 12 to 17 years of age, and the overall rate of tooth fracture experience in the last 12 months was 11.4%. A recent systematic review and meta-analysis by Azami-Aghdash et al. [16] that included 44 studies reported an average tooth fracture prevalence of 17.5% within the age group of 6–18 years. Thus, the tooth fracture experience rate of 11.4% in this study was not higher than that reported previously. Although the tooth fracture experience rate was investigated for 12 months in the present study, it was considered to be lower than that in 11–13-year-olds in Thailand (35.0%) [5] and in Newham, London, UK (43.8%) [6].

The tooth fracture experience rate was higher in males than in females. In terms of health-risk behaviors, a higher average alcohol intake volume and higher number of cigarettes smoked over the last 30 days corresponded to higher rates of tooth fracture. A study in Brazil also reported higher rates of dental trauma among teenagers drinking alcohol [16]. Alcohol consumption is considered to be associated with a high incidence of accidents due to poor behavior control. In addition, Kim and Bea [17] analyzed data from the 7th Korea National Health and Nutrition Examination Survey and reported that the rate of dental trauma experience was high in people who smoked. However, the mechanisms underlying the relationship between smoking and the occurrence of dental trauma have not been reported and are a topic of future research.

In mental health domains, tooth fracture experience was higher in adolescents with higher perceived stress levels, lower fatigue recovery level by sleep, depression, and suicidal ideation experience. Jang and Kim [18] analyzed the relationship between oral and maxillofacial trauma and stress in male students majoring in sports, and had found no significant associations. On the other hand, Lee [19] reported that psychological and emotional factors could potentially play a role in tooth trauma in children and adolescents. Jones [20] considered that a stressful environment may be created when children continue to suffer accidents. Psychological stress has been reported to be one of the factors that can cause accidents in adult occupation activities [21]. Adolescents in Korea are more severely stressed than individuals of any other population group, and excessive stress weakens the social and psychosocial functions of adolescents [22]. This phenomenon may be a potential factor influencing accidents including dental trauma. Sleep is the primary means of fatigue recovery. However, inadequate fatigue recovery due to lack of sleep can induce problems with cognition, resulting in failure to respond quickly to external reactions or in reducing requisite movements [23,24]. Bicycle riding accidents and slips and falls in classrooms, corridors, playgrounds, toilets, stairs, and other unspecified situations show positive correlations with sleep deprivation [25]. Therefore, insufficient fatigue recovery due to lack of sleep can increase risk of accidents of the facial region, causing more tooth fractures.

Depression is assessed by determining whether the individual has felt sad or desperate enough to stop his/her daily life over the previous two weeks. In a study of adolescents in eastern London, depression experience was reported to show no significant relationship with the rate of dental trauma [26]. However, in another study, 31-year-olds who experienced depression in northern Finland reported a higher rate of dental trauma than those who did not [13]. Thus, it is necessary to study the relationship of dental trauma, including tooth fracture, with depression and various psychosocial factors. Adolescents with suicidal ideation can easily feel depressed and are more prone to accidents because of difficulties in paying attention [27]. Thus, tooth fractures may also occur due to injuries caused by distractions.

In the physical exercise domain, the relationship between the number of days on which intense physical exercise was performed and the rate of tooth fracture experience was confirmed. In the questionnaires, “jogging, soccer, basketball, taekwondo, mountain climbing, fast cycling, fast swimming, carrying heavy objects, etc.” were suggested as examples of intense physical exercise. Intense physical exercise was likely to involve damage due to face-to-face contact with a person or object. Werlich et al. [28] performed a systematic review and literature analysis and reported that the incidence of tooth and facial injuries was 27.57% in contact sports players, with incidences of 37.36% in rugby, 27.26% in basketball, 24.58% in handball, and 19.0% in field hockey. Among maxillofacial injuries, incidence of dental trauma was reported to be 19.6%. A systematic review and meta-analysis by Polmann et al. [29] determined that the overall pooled prevalence of dentofacial injuries in combat sports was approximately 30%. The prevalence of dentofacial injuries was the highest in jiu-jitsu (52.9%) and the lowest in judo (25.0%), and Pan-American sports reported the highest prevalence of dental injuries (73.7%) in boxing.

Dental trauma, including tooth fracture, is most common in the maxillary anterior region [29]. Therefore, a mouth guard is recommended to prevent tooth injuries [30,31]. The first sport to mandate use of a mouth guard was professional boxing in 1920, and the USA National Collegiate Athletic Association mandated the use of a mouth guard in four sports (ice hockey, lacrosse, field hockey, and football). The American Dental Association recommends use of a mouth guard in 29 sports [32]. However, due to the discomfort associated with wearing mouth guards or difficulties in breathing, only a small number of sports players wear mouth guards in Korea, so more practical mouth guards should be developed and recommended [30,31,33].

Adolescents who performed more days of muscle strength exercise showed a higher rate of tooth fracture experience. These exercises aim to strengthen cardiopulmonary function and increase muscle mass, and while some muscle strength exercises do not use equipment, others require exercise equipment. Causes of injury in exercise include factors related to training, body, or physical condition (imbalance of muscle strength, lack of flexibility, and mental anxiety), and environmental factors (protective equipment and assessment of exercise environment) [34]. Since the primary focus at present is prevention and treatment of sports damage [32,35], research on the relationship between muscle strength exercise and tooth trauma is rare, and use of mouth guards is recommended to prevent tooth and facial damage [31,36].

Thus, considering the overall study findings, risk factors related to tooth fracture in adolescents include health-risk behaviors such as alcohol consumption and smoking, mental health factors such as stress perception, depression, and suicidal ideation, and physical exercise-related factors such as days of intense physical exercise and muscle strengthening exercise. The most relevant variable for tooth fracture experience was depression. Depression among adolescents will affect overall daily life, and this study confirmed that it is also significant for tooth fractures. Thus, interventions for mental health promotion to relieve depression and mental stress in adolescents are essential. In addition, use of a mouth guard when performing vigorous exercise in school sports activities should be encouraged and the school environment should focus on preventing tooth fractures due to facial injuries in school. This study only analyzed the questionnaire assessments for variables that cause tooth fracture. However, environmental factors, including those within and outside the school, were not reviewed in this study. Since factors encountered in field training in vocational high schools were not reviewed, further studies are warranted. Nevertheless, the results of this study analyzing factors responsible for adolescent dental fractures using Korean national representative data can help design interventions to prevent tooth fractures.

## 5. Conclusions

The overall rate of tooth fracture experience in the last 12 months among adolescents was 11.4%. Risk factors for tooth fracture in adolescents included health-risk behaviors such as alcohol consumption and smoking, mental health factors such as stress perception, depression, and suicidal thoughts, and physical exercise factors such as days of intense physical exercise and muscle strengthening exercise. The most relevant variable for tooth fracture experience was depression. Thus, it is necessary to prepare interventions for mental health promotion to relieve depression and mental stress in adolescents. In addition, use of a mouth guard during vigorous exercise in school sports activities as well as development of a school environment to prevent tooth fractures due to facial injuries in school are important measures.

## Figures and Tables

**Table 1:** Health-risk behaviors and mental health and physical exercise factors related to tooth fracture experience from the 14th Korea Youth Risk Behavior Survey in 2018.

Domain	Duration	Variables
Alcohol intake	Past 30 days	Mean alcohol intake Cycle of binge drinking experience
Tobacco	Past 30 days	Mean daily tobacco consumption
Mental health	Usually	Usual stress perception level
	Past 7 days	Fatigue recovery level by sleep
	Past 12 months	Depression experience, suicidal ideation experience
Physical activity	Past 7 days	Days of >60-min physical exercise, days of intensive physical exercise, days of muscle strength exercise, days of school gym class

**Table 2:** Tooth fracture experience related to health-risk behaviors and mental health status.

Risk Behaviors and Mental Health	Tooth Fracture Experience (%)	*p* Value
Mean alcohol intake *		<0.001
Never ^†^	10.7	
≤1 bottle	11.8	
2 bottles	16.4	
3 bottles	16.1	
4 bottles	17.0	
≥8 bottles	20.4	
Binge drinking experience ^‡^		<0.001
Never ^†^	11.2	
1–2 days	17.3	
3–4 days	19.1	
5+ days	24.8	
Mean daily tobacco consumption ^§^		<0.001
Never ^†^	10.9	
<1	18.4	
1	17.0	
2–5	17.5	
6–9	18.3	
10–19	23.8	
≥20	22.9	
Usual stress perception ^∥^		<0.001
So much	14.3	
Much	12.4	
A little	10.7	
Less	9.6	
Never	10.9	
Fatigue recovery by sleep ^¶^		<0.001
More than enough	10.7	
Enough	10.4	
Fair	10.9	
Not enough	11.6	
Never	14.0	
Depression ^a^		<0.001
Inexperience	10.3	
Experience	14.5	
Suicidal ideation ^a^		<0.001
Inexperience	11.0	
Experience	14.7	

* Volume by beer. ^†^ Never experienced in a lifetime. ^‡^ Cycle. ^§^ Number who smoke cigarettes. ^∥^ Level. ^¶^ Level in the past 7 days. ^a^ In the past 12 months. *p* value was determined by complex-sample chi-square test.

**Table 3:** Adolescents with tooth fracture experience in the past 12 months.

Grade	Total		Male		Female		p *
	N	%	N	%	N	%	
All	60,040	11.4	30,463	11.8	29,577	11.1	0.023
Middle school							
Grade 1	9847	11.0	4960	11.8	4887	10.1	0.011
Grade 2	10,092	11.2	5137	11.4	4955	10.9	0.505
Grade 3	10,290	11.5	5231	11.3	5059	11.7	0.611
High school							
Grade 1	9260	11.4	4805	12.0	4455	10.8	0.080
Grade 2	10,039	10.9	5110	11.2	4929	10.5	0.251
Grade 3	10,512	12.5	5220	12.7	5292	12.4	0.626
*p* value *		0.009		0.207		0.007	

* Complex-sample chi-square test. N: unweighted value, %: weighted value. Age of adolescents: grade 1 of middle school, 12 years; grade 1 of high school, 15 years.

**Table 4:** Tooth fracture experience related to physical exercises.

Physical Activities *	Tooth Fracture Experience (%)	*p* Value
Days of ≥60-min physical exercise		<0.001
None	10.8	
1 day	12.1	
2 days	10.7	
3 days	11.6	
4 days	10.7	
5 days	12.7	
6 days	11.4	
7 days	15.1	
Days of intensive physical exercise		<0.001
None	10.6	
1 day	11.3	
2 days	11.0	
3 days	11.2	
4 days	11.7	
≥5 days	13.6	
Days of muscle strength exercise		<0.001
None	10.5	
1 day	11.8	
2 days	12.3	
3 days	11.8	
4 days	12.2	
≥5 days	13.6	
Number of school gym classes		<0.001
None	11.6	
1	11.4	
2	10.7	
≥3	12.1	

* frequency in the past 7 days. *p* value was determined by complex-sample chi-square test.

**Table 5:** The adjusted association of tooth fracture experience related to health-risk behaviors by complex-sample multivariable logistic regression models.

Variables	Model 1	Model 2
	Adjusted OR (95% CI)	Adjusted OR (95% CI)
Sex (Ref. = Female)		1.10 (1.04–1.17)
Grade		0.98 (0.096–1.00)
Mean alcohol intake *	1.17 (1.14–1.19)	1.10 (1.07–1.13)
Binge drinking experience *	1.42 (1.31–1.54)	1.02 (0.93–1.12)
Mean number of daily cigarettes consumed *	1.18 (1.16–1.21)	1.10 (1.07–1.13)
Usual stress perception level (Ref. = Never)	1.15 (1.12–1.18)	1.06 (1.03–1.10)
Fatigue recovery level by sleep ^†^ (Ref. = More than enough)	1.09 (1.07–1.12)	1.03 (1.00–1.06)
Depression experience ^‡^ (Ref. = No)	1.49 (1.41–1.57)	1.29 (1.21–1.37)
Suicidal ideation experience ^‡^ (Ref. = No)	1.42 (1.33–1.52)	1.09 (1.01–1.18)

* In the past 30 days, ^†^ In the past 7 days, ^‡^ In the past 12 months. Dependent variable: tooth fracture experience (reference category = inexperience). Model 1: adjusted for sex and grade. Model 2: adjusted for sex, grade, usual stress perception, fatigue recovery by sleep, depression, suicidal ideation, mean alcohol intake, binge drinking experience, and mean daily tobacco consumption.

**Table 6:** The adjusted association of tooth fracture experience related to physical exercise by the complex-sample multivariable logistic regression models.

Variables	Model 1	Model 2
	Adjusted OR (95% CI)	Adjusted OR (95% CI)
Sex (Ref. = female)		0.98 (0.92–1.04)
Grade		1.02 (1.01–1.04)
Days of >60-min physical exercise	1.03 (1.02–1.05)	1.01 (0.99–1.03)
Days of intensive physical exercise	1.05 (1.03–1.07)	1.03 (1.00–1.05)
Days of muscle strength exercise	1.06 (1.04–1.07)	1.04 (1.02–1.06)
Days of school gym class	1.02 (1.00–1.05)	0.99 (0.97–1.02)

Dependent variable: tooth fracture experience (reference category = inexperience). Model 1: adjusted for sex and grade. Model 2: adjusted for sex, grade, days of >60-min physical exercise, days of intensive physical exercise, days of muscle strength exercise, and days of school gym class.
